# Initial findings in traumatic peripheral nerve injury and repair with diffusion tensor imaging

**DOI:** 10.1002/acn3.51270

**Published:** 2021-01-06

**Authors:** Michael D. Pridmore, Gabriella E. Glassman, Alonda C. Pollins, Isaac V. Manzanera Esteve, Brian C. Drolet, Douglas R. Weikert, Mark D. Does, Galen Perdikis, Wesley P. Thayer, Richard D. Dortch

**Affiliations:** ^1^ Vanderbilt Institute for Imaging Science Vanderbilt University Medical Center Nashville Tennessee USA; ^2^ Department of Plastic Surgery Vanderbilt University Medical Center Nashville Tennessee USA; ^3^ Department of Orthopaedic Surgery Vanderbilt University Medical Center Nashville Tennessee USA; ^4^ Department of Biomedical Engineering Vanderbilt University Nashville Tennessee USA; ^5^ Department of Neuroimaging Research Barrow Neurological Institute Phoenix Arizona USA

## Abstract

**Objective:**

Management of peripheral nerve injuries requires physicians to rely on qualitative measures from patient history, electromyography, and physical exam. Determining a successful nerve repair can take months to years for proximal injuries, and the resulting delays in clinical decision‐making can lead to a negative impact on patient outcomes. Early identification of a failed nerve repair could prevent permanent muscle atrophy and loss of function. This study aims to test the feasibility of performing diffusion tensor imaging (DTI) to evaluate injury and recovery following repair of wrist trauma. We hypothesize that DTI provides a noninvasive and reliable assessment of regeneration, which may improve clinical decision‐making and alter the clinical course of surgical interventions.

**Methods:**

Clinical and MRI measurements from subjects with traumatic peripheral nerve injury, carpal tunnel syndrome, and healthy control subjects were compared to evaluate the relationship between DTI metrics and injury severity.

**Results:**

Fractional anisotropy from DTI was sensitive to differences between damaged and healthy nerves, damaged and compressed nerves, and injured and healthy contralateral nerves. Longitudinal measurements in two injury subjects also related to clinical outcomes. Implications of other diffusion measures are also discussed.

**Interpretation:**

DTI is a sensitive tool for wrist nerve injuries and can be utilized for monitoring nerve recovery. Across three subjects with nerve injuries, this study has shown how DTI can detect abnormalities between injured and healthy nerves, measure recovery, and determine if re‐operation was successful. Additional comparisons to carpal tunnel syndrome and healthy nerves show that DTI is sensitive to the degree of impairment.

## Introduction

Peripheral nerve damage following injury can result in catastrophic clinical outcomes if not detected and treated in a timely manner. Etiologies of peripheral neuropathy can be divided into traumatic and nontraumatic causes. Traumatic peripheral nerve injury (TPNI) typically has a devastating impact on function and quality of life. TPNI may result in temporary or permanent paralysis, irreversible muscle atrophy, and/or formation of painful neuromas.^1^ TPNI occurs in 3‐5% of Level 1 trauma cases and results in approximately 100,000 operative procedures in North America annually,^2^, ^3^ with many additional cases noted in recent military missions.^4^, ^5^ Literature has shown^1^, ^2^, ^6^, ^7^ that TPNI affects the upper extremity more frequently than other areas.

Neurotmesis in the upper extremity is a common, but potentially devastating, injury wherein all components of a nerve (axon, endoneurium, perineurium, and epineurium) are completely transected. This significant injury warrants surgical repair to re‐establish proximal and distal connections. Successful recovery requires axonal regeneration from the repair site to the target tissue at a growth rate of approximately one inch per month.^1^ Nerve transfers can reduce the length of axonal growth required to reach target tissue, and nerve grafts provide a conduit to create less tension on the repair; however, approximately 40% of surgical repairs fail following these injuries.^8^, ^9^ Clinical management following a TPNI generally entails ongoing clinical evaluation combined with electrodiagnostic testing. This “watch and wait” methodology takes weeks to months, which can be frustrating for patients and their physicians. Using this management, failure of nerve regeneration often goes unnoticed for extended periods, during which the window of opportunity for a revisional surgery closes, leading to irreversible muscle atrophy due to prolonged muscle denervation.^10^

Current diagnostic tools following a traumatic extremity injury are limited to clinical examinations and electrodiagnostic tests.^11^ In particular, nerve conduction studies (NCS) and electromyography (EMG) remain the mainstay for defining the severity and distribution of motor and sensory function following a TPNI. However, reinnervation typically does not begin until 3–6 weeks following an inciting event. During regeneration, NCS and EMG together provide an incomplete picture of nerve pathology.^12^ Additionally, these tests often fail to discriminate neurotmesis or axonotmesis, involving division of axons and elements of the epineurium, from a mild self‐resolving neuropraxic injury,^11^, ^12^, ^13^, ^14^, ^15^ such as compression, in the acute setting prior to surgical intervention.

Given the time‐sensitive nature of nerve regeneration, high‐resolution ultrasonography (HRUS) and magnetic resonance neurography (MRN) imaging have been proposed to mitigate current diagnostic limitations and to explore mapping of peripheral nerve regeneration. HRUS can accurately identify transected nerves,^16^, ^17^ but its use can be limited in traumatic injuries due to associated hematomas, edema, skin lacerations, and alterations of the normal anatomy.^16^ Conventional magnetic resonance imaging (MRI) of nerves allows for both longitudinal and cross‐sectional fascicular images to detect nerve discontinuity, localize nerve compression, and decipher intraneural from perineural masses.^18^ Areas affected by peripheral neuropathy demonstrate hyperintense changes on MRI T_2_‐weighted sequences that can characterize myelin and axonal health; however, MRN alone cannot discriminate between myelin and axonal pathologies and, therefore, lacks specificity.^19^

Diffusion tensor imaging (DTI) is a quantitative MRI method that has been particularly effective in detecting and monitoring central nervous system (CNS) pathologies such as traumatic brain injury, spinal cord injury, and multiple sclerosis.^20^, ^21^, ^22^ This method has also shown promising results in the detection of myelin and axon pathology for peripheral nerves like those in the wrist.^23^, ^24^ DTI has previously been used to detect peripheral nerve regeneration as early as 1–3 months in humans.^25^, ^26^, ^27^ DTI provides a noninvasive, quantitative approach to evaluate tissue microstructures throughout the recovery process by measuring the diffusion of water molecules in tissue over multiple directions.^28^ In the absence of barriers, diffusion is isotropic or equal in all directions. Conversely in ordered, elongated biological tissue like axons, water diffusion is anisotropic due to its interactions with surrounding tissue structures and cellular membranes, resulting in an apparent diffusivity that is the highest along the primary direction of the axons.^25^, ^28^, ^29^

Given this sensitivity to nerve microstructure, we aimed to determine the sensitivity of DTI in monitoring nerve degeneration and regeneration following injury and surgical repair. In previous animal studies, we and others demonstrated that DTI could successfully identify and grade complete and partial transections of sciatic nerves in ex vivo rats.^30^, ^31^ In this study, we employed DTI on distal nerves of the wrists of human subjects to longitudinally evaluate nerve repair and regeneration. In addition, we included a cohort of carpal tunnel syndrome (CTS) patients to compare the sensitivity of this method to less severe, compression injuries. If successful, these DTI strategies could help improve patient outcomes by predicting regenerative failures earlier than current techniques, and even provide support for early re‐operation.

## Methods

### Human subjects and clinical information

Three subjects with TPNI, eight subjects with CTS, and eight healthy control subjects were consecutively enrolled in this study. All TPNI subjects underwent microsurgical nerve repairs of fully lacerated median and/or ulnar nerve(s) at the Vanderbilt University Medical Center (VUMC) by fellowship‐trained hand surgeons. All CTS subjects underwent open carpal tunnel release surgery and were treated with the standard clinical care procedure at VUMC. All healthy controls were volunteers recruited to match the sex and range of ages of the TPNI cohort. Subjects and healthy volunteers were excluded from recruitment if they reported a history of other concurrent neuropathies (e.g., diabetic neuropathies) or wrist trauma. One CTS subject was excluded due to an additional Charcot‐Marie‐Tooth Type 1A diagnosis, and one TPNI subject was excluded due to a radial nerve injury at the thumb, which resided outside our imaging coil coverage.

For TPNI cases, the nerves were examined using standard microsurgical techniques and the damage was identified as follows: transected nerve endings were inspected, edges were refreshed to healthy bulging fascicles, and an epineural repair was performed with 8‐0 nylon sutures, taking care to all align vascular structures to optimize fascicular repair. In carpal tunnel release surgery, an incision was made at the base of the palm radial to the hook of the hamate. Through this incision, surgeons located the transverse carpal ligament (TCL), which lies between the skin and the median nerve in the carpal tunnel. By dividing the TCL, compression of the median nerve was relieved.

As detailed in Table 1, the median nerve was affected by compression in the CTS cohort. In the TPNI cohort, two of the three TPNI subjects had an injury where only one nerve was affected (TPNI 1 and TPNI 3). The other TPNI subject had both the median and the ulnar nerve injured (TPNI 2) on the right arm and was dual‐enrolled in another clinical trial at VUMC in which a sealant material composed of polyethylene glycol (PEG) and hypotonic calcium‐free saline was applied directly to the repaired nerve adjunctive to the microsurgical repair. PEG has demonstrated safety in human usage^32^ and PEG fusion has proven effective in terms of functional outcomes and speed of nerve recovery in human digital nerves when compared with standard nerve repair.^33^, ^34^

More specifically, TPNI 1 presented with a linear laceration of the median nerve in the dominant right arm. Along with repair of the median nerve, surgical repair was done on the complete transections of the flexor carpi radialis tendon, the palmaris longus tendon, the flexor pollicis longus tendon, and partial transection of the flexor distal phalanx tendon. TPNI 2 presented with full transection of the median nerve and partial transection of the ulnar nerve in the dominant right arm. Concomitant with this trauma was a flexor tendon injury, which was also repaired. TPNI 2 received PEG gel as part of the median nerve repair. TPNI 3 presented with partial transection of the ulnar nerve on the nondominant left arm. Along with nerve trauma, TPNI 3 had damaged the flexor carpi ulnaris and flexor digitorum superficialis tendons. Additionally, carpal tunnel release surgery was performed on the median nerve as part of the repair procedure. After clinical information from the second timepoint indicated that the initial repair surgery was not successful, TPNI 3 was re‐operated on and a 70 x 5 mm cadaveric nerve graft (Avance) was used to promote repair.

**Table 1:** Demographic information and clinical scores for all subjects. This includes Michigan Handedness Questionnaire (MHQ) and 9‐hole Peg Test (9‐HPT). Scores of N/A indicate where measures were not obtained. Scores listed as "unable" may also be considered a score of zero. Multiple scores for TPNI 2 and TPNI 3 reflect measures gained at subsequent clinical visits. R = Right arm and L = Left arm. M = Median nerve and U = Ulnar nerve. Time listed in months since surgery

Subject	Demographics:			Time since surgery:	MHQ:		Grip test:		9‐HPT:	
	Age (Sex)	BMI	Arm ‐ Nerve	(months)	Injured	Healthy	Injured	Healthy	Injured	Healthy
TPNI 1^2^	26 (F)	19.7	R ‐ M	1	N/A	N/A	N/A	N/A	N/A	N/A
TPNI 2	22 (M)	19.8	R ‐ M	1	33	83.3	Unable	92	Unable	23
			& U	4	69	83.3	30	100	100	27
TPNI 3	52 (M)	31.8	L ‐ U	3	5	75.1	30	75	43	27
				6	0.8	81.5	18	74	51	26
				9^1^	5.8	81.5	N/A	N/A	N/A	N/A
Group:	33.3 (66% M)	31.8								
CTS 1	73 (M)	23.5	R ‐ M	5, 16, 20, 24						
CTS 2	48 (F)	34.4	R ‐ M	4.5, 13.5, 16.5						
CTS 3	58 (F)	26.6	R ‐ M	1, 12, 16						
CTS 4	40 (M)	26.6	R ‐ M	4, 14						
CTS 5	44 (F)	30.8	L ‐ M	<1, 3.5						
CTS 6	48 (F)	38.4	R ‐ M	1, 14						
CTS 7	52 (F)	32	R ‐ M	6						
CTS 8	68 (M)	31.4	R ‐ M	2						
Group:	53.3 (38% M)	30.5								
Control 1	57 (M)	23.1	R ‐ M							
Control 2	54 (F)	32.7	R ‐ M							
Control 3	23 (M)	23.8	R ‐ M							
Control 4	27 (M)	19.3	R ‐ M							
Control 5	27 (M)	28.8	R ‐ M							
Control 6	20 (M)	26.5	R ‐ M							
Control 7	25 (M)	30.1	R ‐ M							
Control 8	26 (M)	21	R ‐ M							
Group:	32.4 (88% M)	25.7								

^1^ Indicates measures acquired after second surgery in TPNI 3.

^2^ Indicates knee coil was used during MRI acquisition for TPNI 1.

All TPNI subjects underwent an initial MRI one to three months after surgical repair. Additional longitudinal data were collected in three‐month intervals following the initial scans in two of the three TPNI subjects. All CTS subjects had postoperative MRI one to five months after carpal tunnel release surgery, with the exception of one subject who had preoperative MRI less than a month before surgery. Additional longitudinal data were collected in six of the eight CTS subjects when available. Additional clinical assessments were conducted on TPNI subjects, including: Michigan Handedness Questionnaire (MHQ), Grip test, and 9‐hole Peg Test (9‐HPT). Clinical assessments for CTS subjects are not reported. During the consenting process, all subjects self‐reported no symptoms that were suggestive of peripheral nerve disease and physical examination indicated no evidence of any signs of other peripheral neuropathies. Each subject’s demographic information and clinical scores (when available) are reported in Table 1.

### Standard protocol approvals, registrations, and patient consents

The study was approved by our local Institutional Review Board and all participants provided informed consent prior to all examinations.

### Data availability statement

Deidentified data related to this study will be made available from the corresponding author upon request.

### MRI data acquisition

Subjects were imaged in the prone position with one arm extended above the head (i.e., “superman” position) with a 3.0‐T Philips Ingenia MR scanner. An 8‐channel wrist coil was used for RF reception in all subjects except one, where a larger 16‐channel knee coil was used due to a cast covering the arm. In each MRI session for TPNI subjects, the arm with the most recent injury and surgery was scanned followed by the contralateral, uninjured arm as an internal control. For CTS subjects, three out of the eight subjects had bilateral CTS, therefore the arm with the most recent surgery was reported in our results to ensure consistency across CTS subjects. Healthy subjects had only one arm scanned, as there was no expectation to have differences between healthy nerves.^35^ MR images were acquired in both proximal and distal areas from the injury site, covering from mid‐forearm to the base of the palm. More specifically, for TPNI subjects imaging was not centered over the region of transection (i.e., the center slice is not the injury site). This was due to limited coil coverage, heterogeneity of injuries, and uncertainty of anatomical landmarks to where injury occurred. Nevertheless, these varying injuries in TPNI subjects were captured within our imaging volume in all subjects. A single‐shot EPI scan with diffusion‐weighting was performed at slice thicknesses of 4‐mm with 10‐16 slices in each subject. Additional parameters included: resolution = 0.75 x 0.75 x 4 mm^3^ (wrist coil) or 1.25 x 1.25 x 8 mm^3^ (knee coil), TR/TE = 3000/53 ms, number of acquisitions = 12, 16 directions, max b‐factor of 800 s/mm^2^, and scan time ≈ 11 min.

### MRI data analysis

MATLAB (The Mathworks, Inc., Natick, Massachusetts, USA) was used for tensor estimation and image registration. Regions of interest (ROIs) were manually selected using the MIPAV^36^ imaging software on all slices for the injured and contralateral nerve(s), and mean slice‐wise values were estimated for fractional anisotropy (FA = 0‐1 with increasing values indicating higher anisotropy), mean diffusivity (MD, mean diffusivity in all directions), radial diffusivity (RD, diffusivity perpendicular to axons), and axial diffusivity (AD, diffusivity parallel to axons). Due to the inherent distortion of EPI data and the lack of robust nonrigid registration tools for upper extremity applications, ROIs were drawn directly on the diffusion data. To minimize bias, these ROIs were drawn on the nondiffusion‐weighted (b0) image rather than the parametric maps. The resulting ROI values are reported in Table 2.

**Table 2:** Results of DTI metrics for each subject at each timepoint for each nerve. N/A refers to no surgery. CTS 5 had a diagnosis of carpal tunnel syndrome but had not yet received treatment at first scan. The units of mean diffusivity (MD), axial diffusivity (AD), and radial diffusivity (RD) are μm^2^/ms. Time listed in months since surgery

Subject	Time Postsurgery	State (Nerve)	FA ± SD	MD ± SD	AD ± SD	RD ± SD
TPNI 1^2^	1 month	Injured (Ulnar)	0.45 ± 0.11	1.08 ± 0.23	1.72 ± 0.31	0.77 ± 0.22
	N/A	Contralateral	0.57 ± 0.15	0.92 ± 0.18	1.64 ± 0.26	0.56 ± 0.19
TPNI 2	1 month	Injured (Median)	0.29 ± 0.05	1.39 ± 0.19	1.86 ± 0.24	1.16 ± 0.17
	N/A	Contralateral	0.55 ± 0.13	1.10 ± 0.25	1.90 ± 0.40	0.70 ± 0.20
	4 months	Injured (Median)	0.33 ± 0.07	1.45 ± 0.22	1.99 ± 0.30	1.18 ± 0.20
	N/A	Contralateral	0.53 ± 0.17	1.18 ± 0.34	2.00 ± 0.50	0.77 ± 0.31
	1 month	Injured (Ulnar)	0.33 ± 0.07	1.45 ± 0.17	1.99 ± 0.18	1.18 ± 0.19
	N/A	Contralateral	0.51 ± 0.17	1.12 ± 0.34	1.83 ± 0.49	0.76 ± 0.30
	4 months	Injured (Ulnar)	0.37 ± 0.10	1.36 ± 0.28	1.93 ± 0.34	1.07 ± 0.27
	N/A	Contralateral	0.60 ± 0.18	1.04 ± 0.29	1.93 ± 0.48	0.59 ± 0.23
TPNI 3	3 months	Injured (Ulnar)	0.31 ± 0.07	1.46 ± 0.22	1.97 ± 0.26	1.21 ± 0.22
	N/A	Contralateral	0.41 ± 0.13	1.15 ± 0.30	1.74 ± 0.46	0.85 ± 0.25
	6 months	Injured (Ulnar)	0.17 ± 0.06	1.81 ± 0.27	2.13 ± 0.32	1.65 ± 0.26
	N/A	Contralateral	0.32 ± 0.11	1.28 ± 0.36	1.78 ± 0.47	1.03 ± 0.32
	9 months^1^	Injured (Ulnar)	0.29 ± 0.12	1.29 ± 0.24	1.68 ± 0.27	1.09 ± 0.26
	N/A	Contralateral	0.36 ± 0.08	1.22 ± 0.21	1.75 ± 0.27	0.96 ± 0.20
Group:		Injured	0.32 ± 0.06	1.41 ± 0.15	1.91 ± 0.19	1.16 ± 0.15
		Contralateral	0.48 ± 0.14	1.13 ± 0.28	1.82 ± 0.42	0.78 ± 0.25
CTS 1	5 months	Compressed (Median)	0.45 ± 0.04	1.24 ± 0.08	1.88 ± 0.09	0.92 ± 0.09
	16 months	Compressed (Median)	0.46 ± 0.03	1.20 ± 0.05	1.87 ± 0.05	0.87 ± 0.06
	20 months	Compressed (Median)	0.41 ± 0.03	1.10 ± 0.07	1.59 ± 0.07	0.85 ± 0.08
	24 months	Compressed (Median)	0.46 ± 0.03	1.20 ± 0.05	1.87 ± 0.05	0.87 ± 0.06
CTS 2	4.5 months	Compressed (Median)	0.43 ± 0.03	1.37 ± 0.05	2.04 ± 0.06	1.03 ± 0.06
	13.5 months	Compressed (Median)	0.35 ± 0.02	1.27 ± 0.04	1.77 ± 0.04	1.02 ± 0.05
	16.5 months	Compressed (Median)	0.43 ± 0.02	1.15 ± 0.04	1.74 ± 0.05	0.85 ± 0.04
CTS 3	1 month	Compressed (Median)	0.41 ± 0.02	1.23 ± 0.04	1.84 ± 0.04	0.93 ± 0.05
	12 months	Compressed (Median)	0.43 ± 0.02	1.33 ± 0.04	2.01 ± 0.06	0.99 ± 0.05
	16 months	Compressed (Median)	0.41 ± 0.02	1.20 ± 0.19	1.80 ± 0.28	0.90 ± 0.17
CTS 4	4 months	Compressed (Median)	0.46 ± 0.02	1.23 ± 0.05	1.90 ± 0.07	0.89 ± 0.05
	14 months	Compressed (Median)	0.42 ± 0.04	1.32 ± 0.07	1.95 ± 0.07	1.00 ± 0.08
CTS 5	<1 month	Compressed (Median)	0.51 ± 0.04	1.24 ± 0.08	1.97 ± 0.08	0.88 ± 0.09
	3.5 months	Compressed (Median)	0.43 ± 0.04	1.31 ± 0.06	1.94 ± 0.05	0.99 ± 0.07
CTS 6	1 month	Compressed (Median)	0.44 ± 0.01	1.36 ± 0.02	2.09 ± 0.04	0.99 ± 0.03
	14 months	Compressed (Median)	0.45 ± 0.02	1.28 ± 0.04	1.98 ± 0.06	0.93 ± 0.04
CTS 7	6 months	Compressed (Median)	0.30 ± 0.04	1.47 ± 0.08	1.87 ± 0.10	1.28 ± 0.09
CTS 8	2 months	Compressed (Median)	0.46 ± 0.03	1.23 ± 0.05	1.91 ± 0.04	0.89 ± 0.06
Group:			0.43 ± 0.03	1.27 ± 0.06	1.90 ± 0.06	1.01 ± 0.06
Control 1	N/A	Healthy (Median)	0.54 ± 0.03	1.13 ± 0.04	1.91 ± 0.04	0.74 ± 0.05
Control 2	N/A	Healthy (Median)	0.39 ± 0.03	1.25 ± 0.05	1.82 ± 0.05	0.96 ± 0.06
Control 3	N/A	Healthy (Median)	0.54 ± 0.02	1.07 ± 0.06	1.79 ± 0.05	0.71 ± 0.06
Control 4	N/A	Healthy (Median)	0.61 ± 0.03	1.04 ± 0.05	1.86 ± 0.05	0.62 ± 0.05
Control 5	N/A	Healthy (Median)	0.64 ± 0.03	1.09 ± 0.08	1.99 ± 0.09	0.64 ± 0.08
Control 6	N/A	Healthy (Median)	0.70 ± 0.02	0.95 ± 0.03	1.89 ± 0.04	0.49 ± 0.04
Control 7	N/A	Healthy (Median)	0.64 ± 0.04	1.07 ± 0.04	2.00 ± 0.03	0.60 ± 0.06
Control 8	N/A	Healthy (Median)	0.66 ± 0.03	1.11 ± 0.03	2.10 ± 0.03	0.61 ± 0.04
Group:			0.59 ± 0.03	1.09 ± 0.05	1.92 ± 0.05	0.67 ± 0.05

^1^ Indicates measures acquired after second surgery in TPNI 3.

^2^ Indicates knee coil was used during MRI acquisition for TPNI 1.

### Statistical analysis

All statistical analyses were conducted using MATLAB Statistics and Machine Learning Toolbox (The Mathworks, Inc.). The measures for comparison derived from DTI included FA, MD, RD, and AD. Nonparametric statistical approaches were used in this study’s analysis and raw p‐values are reported given the exploratory nature of this study. Slice‐wise variations in FA were evaluated via a Kruskal–Wallis test to see if healthy nerves in TPNI contralateral arms exhibited significant variations along the length of the nerve. Next, we conducted a cross‐sectional analysis of all cohorts and timepoints to evaluate the effect of injury severity on the observed DTI metrics in nerves via Wilcoxon Rank‐Sum tests. In addition to this, we compared pairwise DTI metrics from injured and contralateral nerves in the same TPNI patients via Wilcoxon Signed‐Rank tests. Finally, we compared FA of injured and healthy nerves in individual TPNI subjects longitudinally (when available) via Wilcoxon Rank‐Sum tests to assess each subjects’ recovery over time.

## Results

### Clinical data

Clinical measures obtained from all subjects in injured and healthy arms are summarized in Table 1. TPNI 1 did not participate in these clinical measures. Clinical findings for TPNI 2 show improvement between timepoints (1/2) across all measures for MHQ (33.0/69.0), Grip test (Unable/30), and 9‐HPT (Unable/100 sec.). TPNI 3 participated in all clinical assessments for the first and second timepoints, but only participated in the MHQ assessment at the third timepoint. Results of TPNI 3 show a decline in all measures between timepoints (1/2) for MHQ (5.0/0.8), Grip test (30/18), and 9‐HPT (43 sec./51 sec.). After re‐operation, the MHQ score rose above the first measure (5.8). CTS subjects received standard care, which did not include additional clinical measurements.

### Representative data in control, TPNI, and CTS

**Figure 1:**
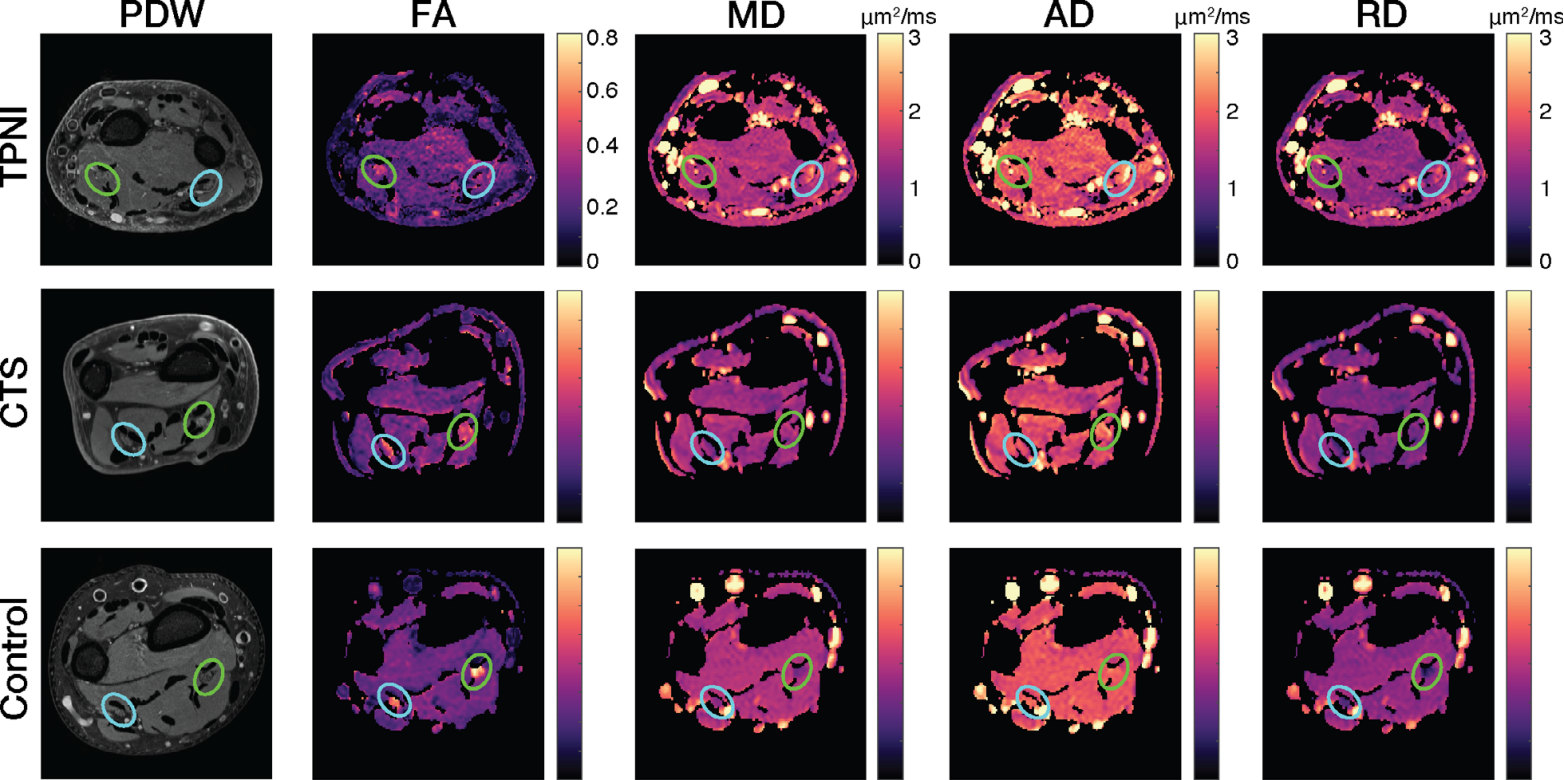
Representative Images: Proton density‐weighted (PDW), fractional anisotropy (FA), mean diffusivity (MD), axial diffusivity (AD), and radial diffusivity (RD) maps show for traumatic peripheral nerve injury cohort (TPNI), carpal tunnel syndrome cohort (CTS), and control cohort. Green circles indicate the median nerve. Blue circles represent the ulnar nerve.

The MRI scanning procedure was well‐tolerated by all subjects. The MR images acquired were artifact‐free from chemical shift due to fat and had very little motion artifacts. In the anatomical and diffusion images, the nerves were easily distinguishable from surrounding tissue (Fig. 1).

As expected, there was no effect of slice position on the DTI values from healthy subjects (*P* = 0.182). As a result, we estimated mean DTI parameters (and 95% confidence intervals from the slice‐wise standard deviation) across slices for the contralateral nerves in each TPNI subject/timepoint, which served as an internal control for comparison to the damaged/repaired ipsilateral nerve in the same TPNI subject and timepoint. In other words, this allowed us to account for demographic factors (e.g., age, sex, body mass index) that may affect results across subjects as well as experimental factors that may affect measurements across time.

### Cross‐sectional analysis across cohorts with different injury severities

**Figure 2:**
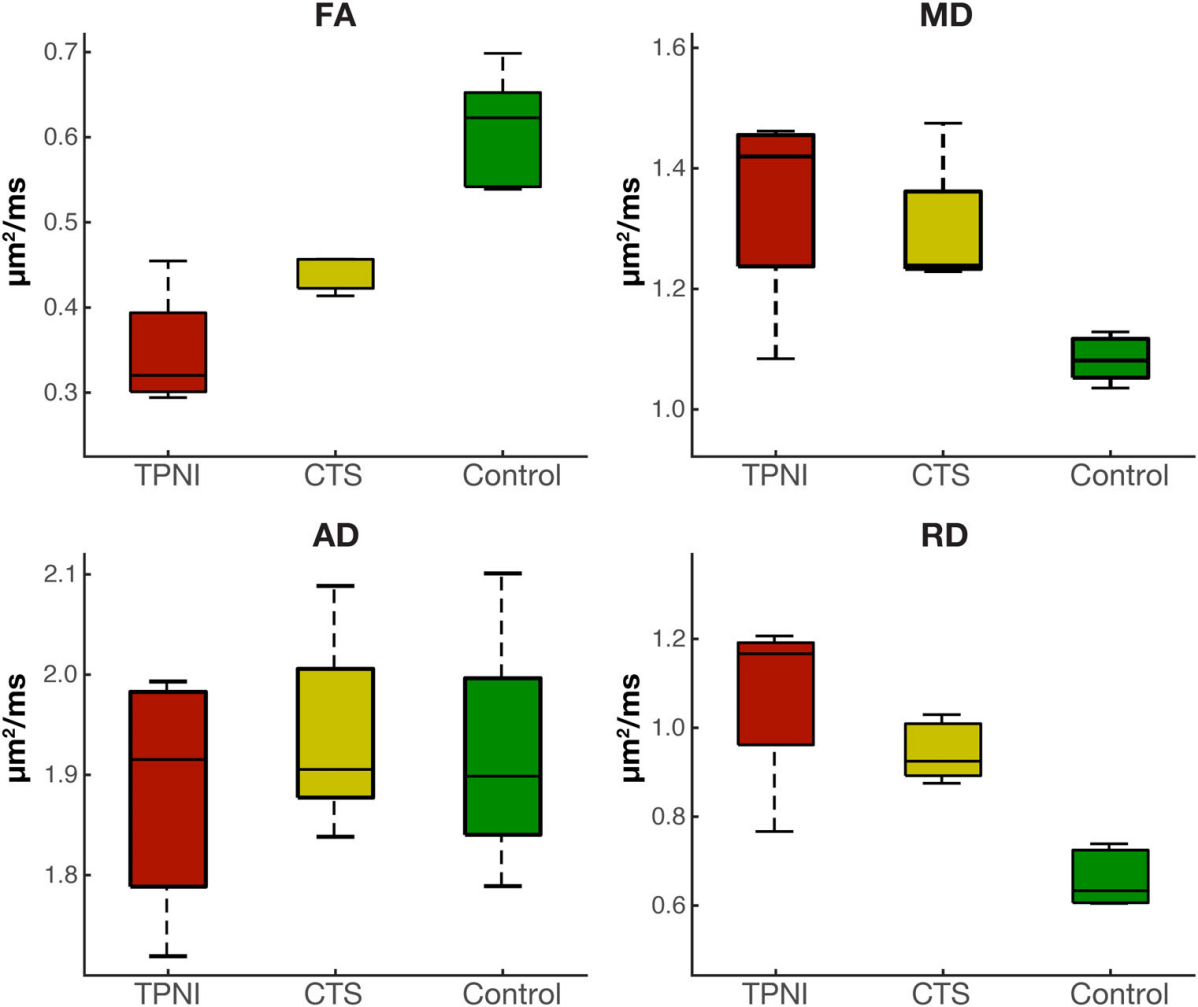
Group comparison of each DTI metric for the traumatic peripheral nerve injury (TPNI: red, *n* = 8), carpal tunnel syndrome (CTS: yellow, *n* = 18), and control cohorts (green, *n* = 8) across all subjects and timepoints. The black central mark represents the median, the edges of the box are the 25th and 75th percentile, and the whiskers show the interquartile range beyond these edges. This includes all longitudinal data for 3 TPNI and 8 CTS subjects.

Figure 2 qualitatively summarizes the full range of values for each cohort, including the postsurgical data across all timepoints in the CTS and TPNI cohorts. Note the overall trend in the range of FA values, which were reduced in the CTS subjects relative to controls, and further reduced in the more severe TPNI subjects relative to the CTS subjects. For the quantitative statistical analysis, control data as well as TPNI/CTS data from each subject’s first timepoint were compared via pair‐wise Wilcoxon Rank‐Sum tests. TPNI data showed no significant differences when compared with CTS for FA (*P* = 0.156), MD (*P* = 0.461), AD (*P* = 0.683), and RD values (*P* = 0.461). However, when compared with controls, TPNI showed significantly decreased FA (*P* = 0.008) and elevated MD (*P* = 0.048) and RD values (*P* = 0.008). Additionally, comparing CTS to controls also showed decreased FA (*P* = 0.007) and elevated MD (*P* = 0.003) and RD values (*P* = 0.003). There were no significant differences in AD for any of these cohort comparisons (*P* > 0.808). It should be noted that while this analysis includes only the first timepoints for both TPNI and CTS groups, there were no significant changes over timepoints after surgery for CTS subjects (Spearman rank correlation coefficient = 0.198, *P* = 0.430). Furthermore, it is worth noting that more variability was present for DTI parameters from TPNI than for the healthy controls, which is likely related to the variability of injuries in this cohort and the combined use of measurements in the proximal and distal slices.

### Cross‐sectional analysis of injured & contralateral nerve in TPNI

**Figure 3:**
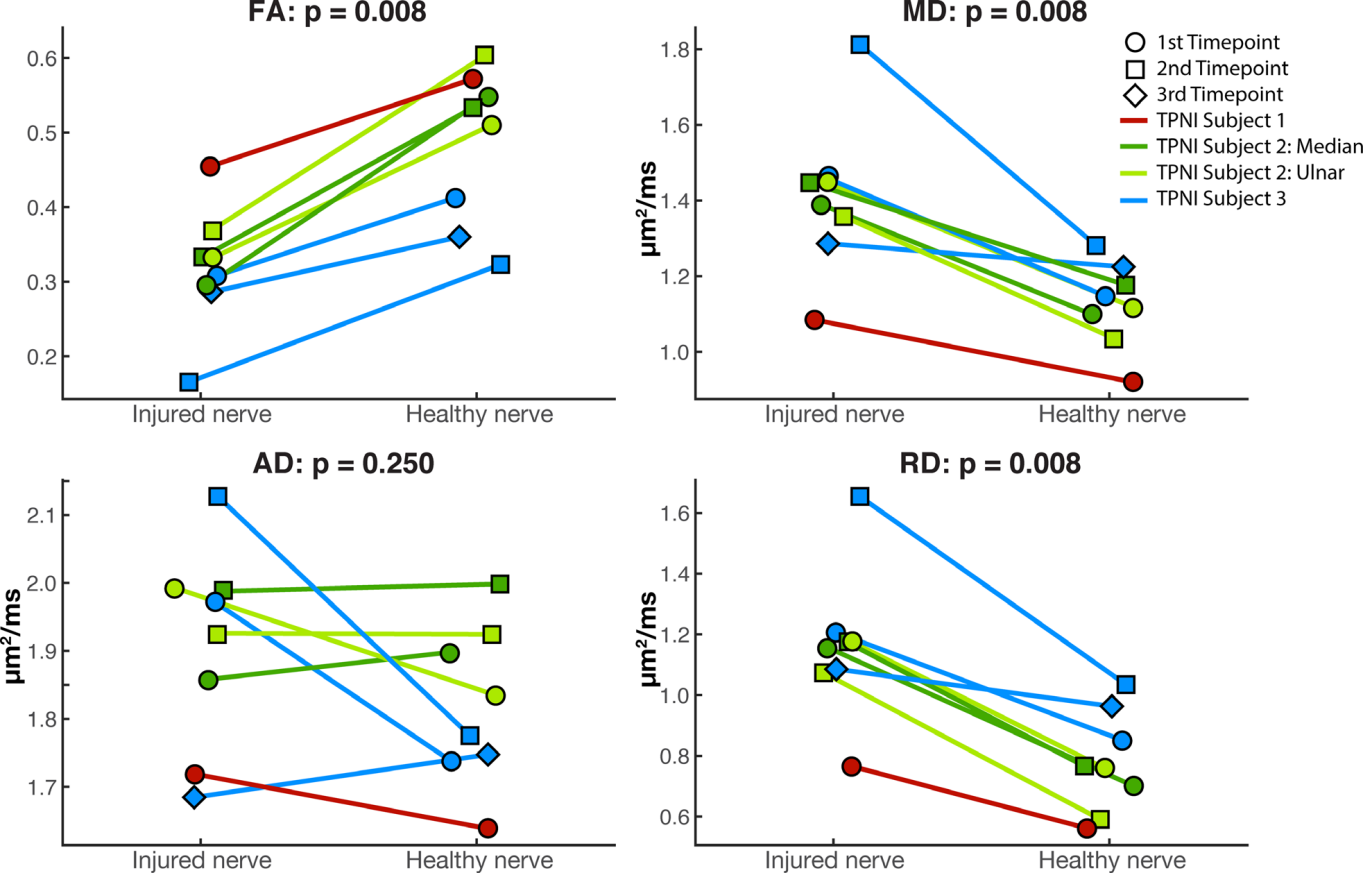
Results of all TPNI subjects (*n* = 3) and timepoints comparing injured nerve data to the contralateral healthy nerve data for all DTI metrics acquired: FA (top‐left), MD (top‐right), AD (bottom‐left), and RD (bottom‐right). Circles represent the first timepoint, squares represent the second timepoint (TPNI 2 & 3), and diamond represents the third timepoint (TPNI 3). Red lines represent TPNI 1, green lines represent TPNI 2 (dark/light = median/ulnar), and blue represents TPNI 3. *P*‐values of Wilcoxon Signed‐Rank test are shown above each boxplot.

TPNI data at each timepoint and nerve injury were used in this analysis, resulting in eight paired data points from three subjects. At the group level, a Wilcoxon Signed‐Rank analysis of DTI metrics showed injured nerves had significantly lower FA values than contralateral nerves (*P* = 0.008), significantly elevated MD (*P* = 0.008) and RD values (*P* = 0.008), and similar AD values (*P* = 0.250). These results are illustrated in Figure 3.

**Figure 4:**
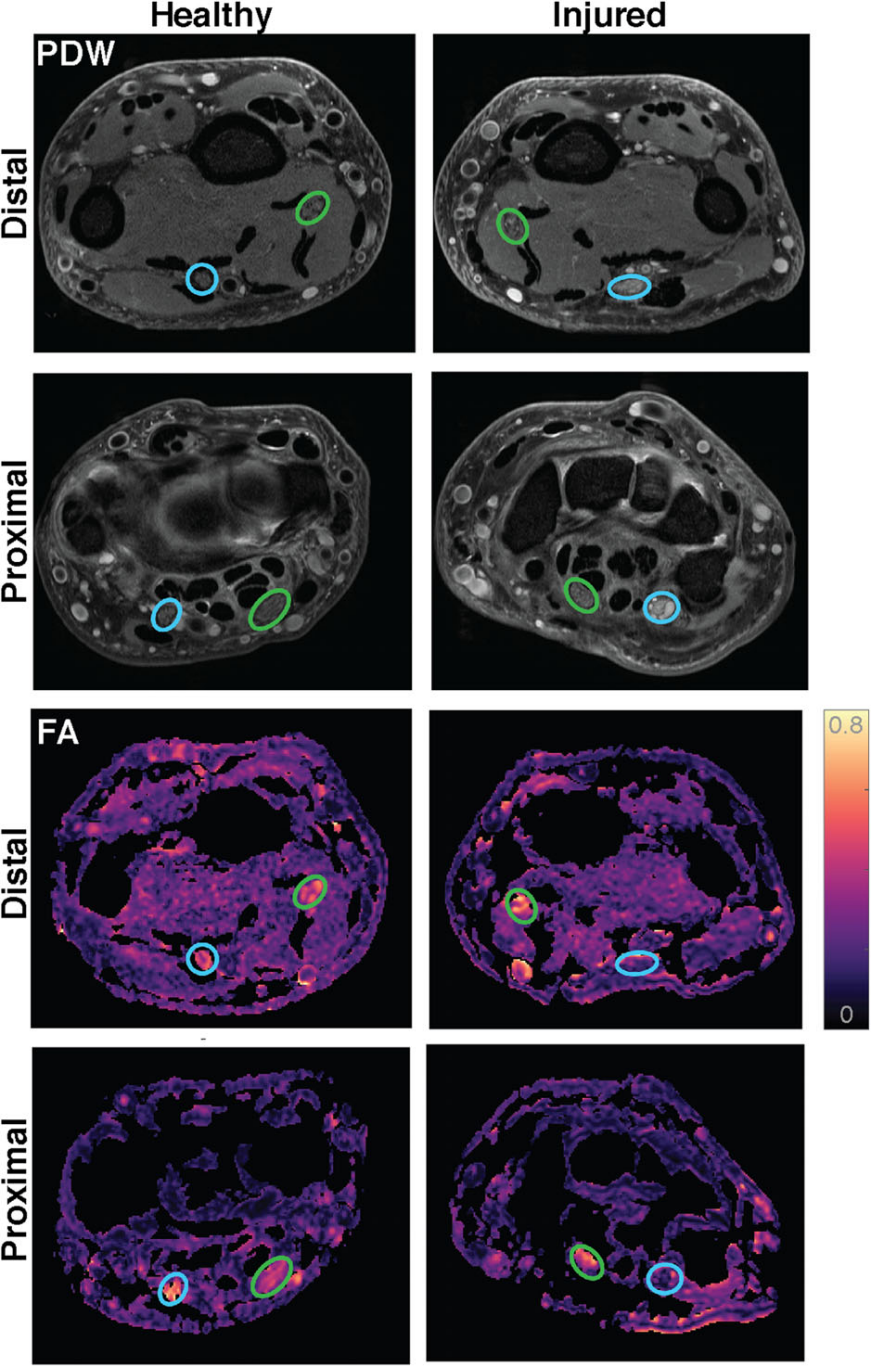
Proton density‐weighted (PDW) and fractional anisotropy (FA) maps for injured and healthy nerves in TPNI 3. Green circles indicate the median nerve. Blue circles represent the ulnar nerve. Worth nothing are the differences in intensity between the injured ulnar nerve and healthy ulnar nerve.

The difference in measures between the injured and contralateral nerve is further illustrated for a single subject in Figure 4, where lower FA values are present in the injured ulnar nerve, and higher FA values in the contralateral ulnar nerve in both proximal and distal images. Note that while Figure 4 shows images of TPNI 3, this phenomenon was observed in the other two TPNI subjects as well.

### Subject TPNI 1

**Figure 5:**
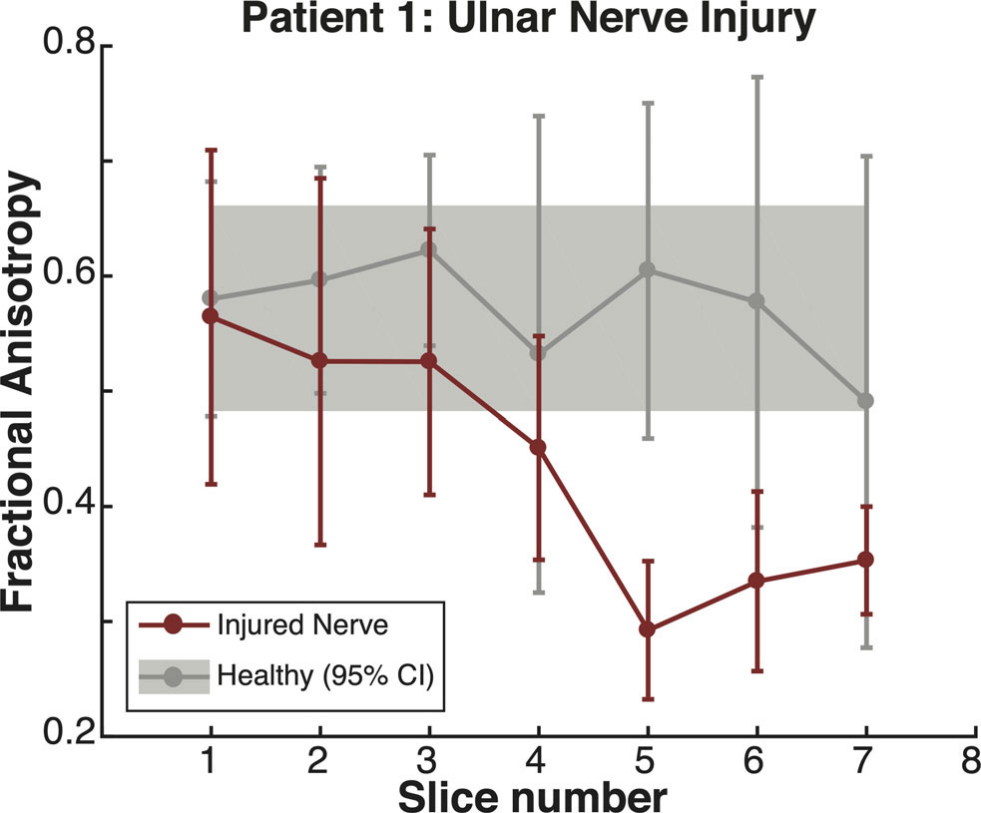
Results for TPNI 1: Fractional anisotropy plotted by slice. Red errorbars reflect individual data points of the injured ulnar nerve, with bars showing slice‐wise standard deviations. Gray errorbars reflect analogous information in contralateral healthy ulnar nerve, with the shaded gray area representing the 95% confidence interval (CI) for averaged healthy ulnar nerve across all slices.

**Figure 6:**
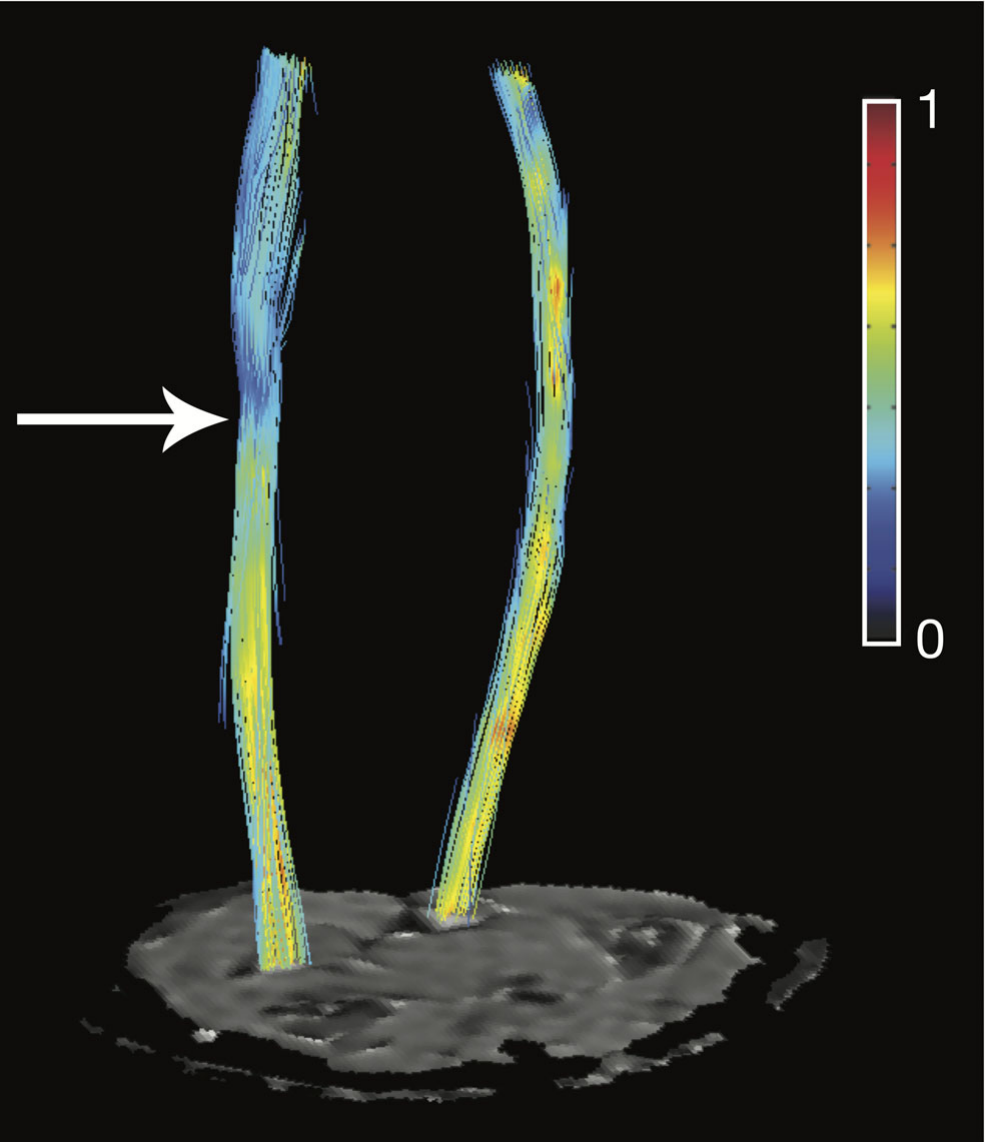
Fibertracking results of TPNI 1 with a right arm median nerve injury. A proximal slice is shown at the bottom of the image with ascending fiber tracking of the injured ulnar nerve (left) and healthy median nerve (right). The white arrow indicates the area of injury. The nerve is color‐coded for fractional anisotropy (FA).

In the first subject, TPNI 1, a comparison of FA between the right arm with the repaired median nerve to the left arm with the uninjured median nerve was shown to be significantly different (*P* = 0.021). Figure 5 shows the slice‐wise measurements for FA in the injured and uninjured median nerves of Subject 1, with more proximal slices (slice 1‐3) of the injured nerve within the boundary of the measures observed in the healthy nerve. As the images move more distal towards the injury site in slices (4‐7), the FA values then fall outside the confidence intervals, with the lowest measure being at slice 6 and continuing upwards with more distal slices. Figure 6 visualizes these data through diffusion fibertracking of the injured median nerve in the right arm, showing the site of injury and how FA values drop distally from the injury toward the hand.

### Subject TPNI 2

**Figure 7:**
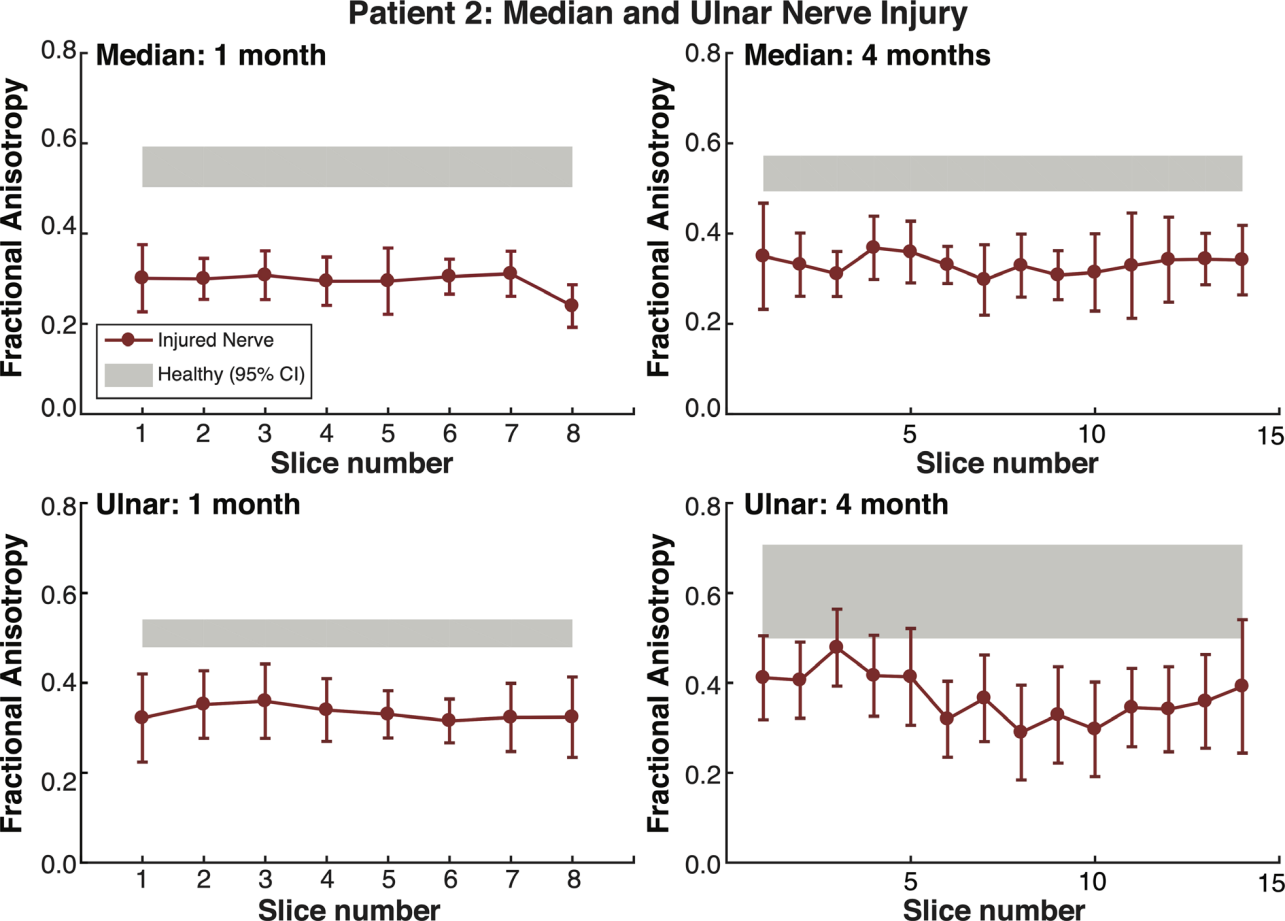
Results for TPNI 2: FA plotted by slice. Red errorbars reflect individual data points of the injured nerves, with bars showing slice‐wise standard deviations. The gray shaded gray area represents the 95% confidence interval (CI) for averaged healthy nerve data in the contralateral arm. The top charts show measures for median nerve across two timepoints. Likewise, the bottom charts show measures for the ulnar nerve across the same timepoints. The ulnar nerve was only partially transected and showed superior recovery, whereas the median nerve was fully transected and showed less recovery by the second timepoint.

In the second subject, TPNI 2, a comparison of the FA of the i) right arm, injured median and ulnar nerves, to the ii) left arm, uninjured median and ulnar nerves, showed significant differences across both longitudinal timepoints (*P* < 0.001, in all cases), with FA being lower in the injured nerve. Figure 7 shows the slice‐wise measurements for FA in the injured and uninjured median and ulnar nerves of TPNI 2. From 1‐month postsurgery to 4 months postsurgery, the injured nerve data fall well below the range of healthy nerve in the contralateral arm; however, there were overall increases of FA in the injured nerve from scan 1 to scan 2, indicating a return toward healthy values over time. It should be noted that this recovery was aided by the PEG treatment. Furthermore, the observed increase in FA across time agreed with the clinical findings in Table 1, which indicated sensorimotor improvement over the injured arm over the same period.

### Subject TPNI 3

**Figure 8:**
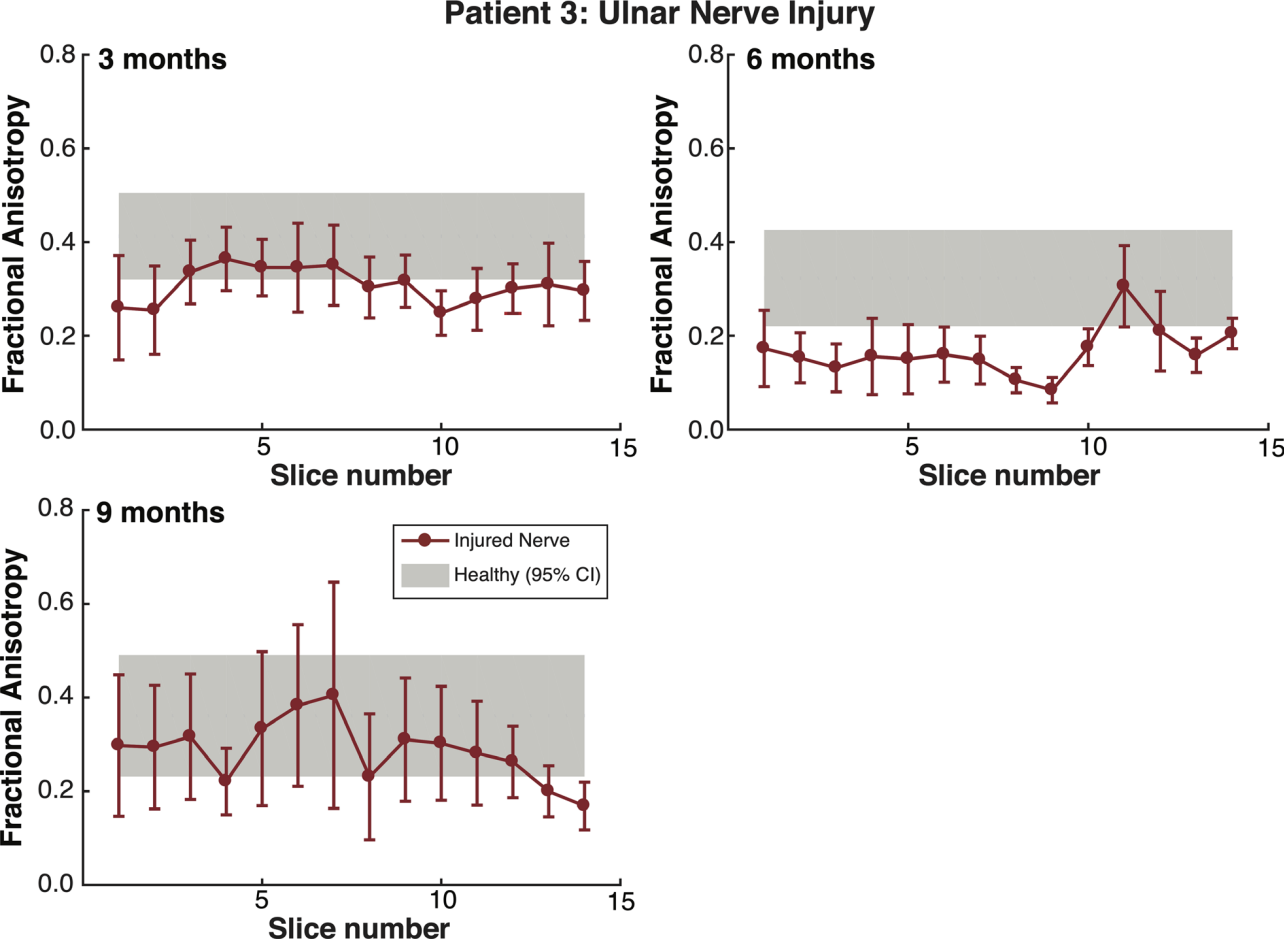
Results for TPNI 3: Fractional Anisotropy plotted by slice. The red errorbars reflect individual data points of the injured ulnar nerve, with bars showing slice‐wise standard deviations. The gray shaded gray area represents the 95% confidence interval (CI) for averaged healthy ulnar nerve data in the contralateral arm. The top charts show measures across two timepoints, 3 and 6 months, whereas the bottom chart shows measures acquired at the third timepoint at 9 months postsurgery. At 6 months, FA decreased which mirrored clinical data showing failure. This led to re‐repair at 9 months and FA recovered.

Once again, FA was significantly lower in injured nerves compared to the contralateral, healthy nerves over all three timepoints in TPNI 3 (*P* < 0.001/*P* < 0.001/*P* = 0.028). Figure 8 shows slice‐wise measures for FA in the injured and contralateral healthy ulnar nerve in TPNI 3. The reduction in FA values at 6 months related to 3 months were consistent with clinical findings reported in Table 1, which indicated an unsuccessful initial repair. Following a secondary repair procedure, FA values overlapped the 95% CI of the contralateral nerve by 9 months (relative to the first procedure), which was once again consistent with clinical findings and is indicative of a successful re‐operation with a nerve graft. In summary, clinical measures showed a decline from the first timepoint to the second timepoint, which agreed with the DTI measures we acquired at these timepoints. In addition to this, clinical measures showed an improvement from the second timepoint to the third timepoint, which indicated the re‐operation between the second and third timepoint was successful, which is reflected in our DTI results as an increased FA. Overall, lower FA were observed in this subject, which exemplifies why having data from the contralateral arm is important in these kinds of studies to correct for potentially confounding demographic factors such as age and body mass index (See: Table 1, TPNI 3).

## Discussion

In this small case–control study, we demonstrated that noninvasive DTI measures are sensitive to traumatic nerve injury and nerve regeneration over time. While the clinical measures do reflect the degree of deficits following TPNI, they are not specific to the degree of injury, which may limit the ability of surgeons to determine when to re‐operate. Across all subjects, i) FA values were significantly reduced in TPNI and CTS cohorts relative to healthy control nerves and ii) DTI findings were consistent with changes in clinical measures across the same periods, suggesting these measures may be indicative of nerve degeneration and regeneration. More specifically, we found that DTI measures improved after a re‐operation procedure (due to a lack of sensorimotor improvement after the first procedure) in one subject (TPNI 3) and after repair‐promoting PEG treatment in another patient (TPNI 2). Together, these findings demonstrate the feasibility of performing DTI in the nerves of the wrist after trauma and surgical repair. Furthermore, these measures may provide a noninvasive and reliable assessment of regeneration, which may improve clinical decision‐making and possibly alter outcomes after surgical interventions.

TPNI 1 suffered a right arm median nerve injury and showed large differences for FA between the injured median nerve compared to the uninjured median nerve of the right arm. These results indicate that DTI can differentiate between injured and uninjured nerves. TPNI 2 suffered a median and ulnar nerve injury at the forearm of the right arm. At the first timepoint, TPNI 2 showed significant differences between both the injured median and ulnar nerves of the right arm when compared with the contralateral uninjured median and ulnar nerves of the left arm. This trend continued three months later at the second timepoint, where significant differences between nerves and arms were observed. For the median nerve, this is conceivably due to the PEG gel that this subject received. For the ulnar nerve, it was expected that the recovery would be more complete, as this nerve experienced a partial transection (the median nerve was fully transected). As these results indicate, DTI can also show improvement over time. TPNI 3 suffered a left arm, ulnar nerve injury that was captured with longitudinal MRI. At the first timepoint, there was a clear delineation between healthy and injured ulnar measures for FA, similar to what was observed in the previous two subjects. At the following timepoint, however, mean FA measures decreased. This agreed with the clinical assessment, which indicated that the initial repair had failed. This subject was brought back to the operating room and a revision was performed. Three months later, FA values increased above what was observed at the first timepoint, indicating that the re‐repair was successful. Therefore, the MRI findings showed that before revision this subject was not healing. The failure rate on nerve repairs is approximately 40%, and this case is an example of how monitoring recovery from nerve trauma can inform the decision to surgically re‐intervene. The ability to identify these failures early, re‐operate, and evaluate over time if the second intervention is working is the ideal application of DTI in monitoring recover after nerve trauma.

These longitudinal findings also indicate that recovery is not homogeneous between subjects. Considering that these traumatic injuries occur in a variety of ways, the rate of recovery can be dramatically different between subjects due to a multitude of factors. Factors such as nutrition, smoking, comorbidities, or, in some cases after injury, overexertion during recovery can slow the healing process. Given this heterogeneity, the objective measures provided by DTI may improve clinical decision‐making by identifying cases that require re‐operation earlier than existing methods, although additional data in larger cohorts are required to evaluate this claim.

### Observed FA changes are driven by RD rather and AD

In these results, it is apparent that RD is the biggest factor affecting the apparent changes in FA in the TPNI subjects. Studies have shown that RD is reflective of myelin integrity and axon density as it measures diffusion perpendicular to the axon, whereas AD is reflective of acute axonal degeneration as it measures diffusion along the axon.^37^, ^38^ As TPNI subjects experience remyelination and axonal sprouting over time, we might anticipate that differences in RD between injured and healthy nerve would arise, as we observed when comparing TPNI with controls (*P* = 0.008), due to the reduced myelination and/or axonal density in recovering nerves. In TPNI, axonal sprouting occurs primarily before secondary remyelination to establish a reconnection from the axon to its target muscle or sensor after damaged tissue has been removed via the process of Wallerian degeneration.^39^ However, it is difficult to separate axonal regeneration from remyelination using measures of RD alone, and a more myelin‐specific measure, like magnetization transfer MRI,^40^ could further improve specificity. For AD, previous animal studies have shown that AD is sensitive to axonal regeneration in the first two weeks after injury.^41^ However, we acquired MRI data later within our cohort (i.e., after Wallerian degeneration), potentially explaining why we did not observe any significant differences for AD between injured and contralateral, healthy nerves (*P* = 0.250).

Another important factor in evaluating the relationship between DTI metrics and underlying tissue microstructure is the influence of inflammation and edema.^42^ In these kinds of injuries, it would be expected to see edema and/or inflammation occur around the injury site, and these effects would diminish over time during the healing process. For these reasons, we would also expect MD to increase with the presence of edema.^43^ The lingering effects of edema over healing may reflect the observed differences we see for MD in comparing TPNI injured and contralateral nerves (*P* = 0.008). In addition, these processes likely also impact FA measurements, as edema is expected to increase diffusion isotropy.

### Comparison to milder injury and healthy controls

The comparison of TPNI, CTS, and healthy subjects showed that DTI was sensitive to the degree of injury across all cohorts. Interestingly, this comparison used the data points acquired from all timepoints, which could also be considered as the range of observed values over the time course for healing. In the TPNI cohort, we saw two of the three subjects improve over time, indicating that the nerve repair was successful to allow for regeneration. However, in the CTS cohort, we did not observe a significant change in FA over time after CTS release surgery. This may be due to the fact the CTS release surgery is not as invasive because the surgery decompresses rather than reconnects detached nerves. In TPNI surgeries, proximal and distal portions of the injured nerve ends are reconnected, with all the damaged tissue removed to allow proper healing. However, in CTS release surgery, only the carpal tunnel ligament is cut, which frees up room around the nerve and releives compression. Additionally, the timepoints we observed CTS subjects may have, in some cases, been after a full recovery, as the time course of full recovery for CTS release surgery is typically 4‐6 months.^44^ Finally, in the control group, higher values for FA than in CTS, which is consistent with recent literature.^45^, ^46^, ^47^, ^48^

### Limitations

It is worth mentioning the limitations of this study for accurate interpretations of the findings. We obtained a small cohort, and as such, chose to analyze the data mostly on an individual subject basis. In addition, the affected nerve (median and/or ulnar), injury severity (full/partial transection), and treatment (PEG, reoperation) varied across TPNI subjects, all of which have a significant impact on outcomes. For these reasons, we expect differences in recovery rate. To overcome these limitations, we analyzed each TPNI subject individually, comparing results in the injured nerve(s) to the contralateral arm on a per subject basis for only the TPNI cohort. In addition, TPNI subjects showed greater variability in contralateral nerve DTI measures, which may be explained by subject comfort diminishing over time and related motion artifacts. This effect was also present in the subset of CTS subjects that received bilateral scans (data not shown). Given these findings, future studies will focus on methods that maximize subject comfort and minimize scan time. For example, we will investigate methods that allow for supine scanning that overcome issues with distortion that are increased in this position. Finally, the CTS and control groups were not age‐ and sex‐matched, which if done correctly would lead to greater generalizable findings and eliminate the possibility of confounding variables. Future work will focus on developing study designs that account for these limitations. Also broadening the timepoints of the study may provide additional information on recovery as traumatic injury and carpal tunnel syndrome recover at different rates. A larger pool of TPNI subjects will also allow us to group by types of injury (e.g., full vs. partial laceration) and by which nerve(s) was damaged (median, ulnar, both). Additionally, clinical measures and presurgical data should be utilized in future studies to evaluate the prognostic value of DTI indices and to better characterize heterogeneity with each cohort (TPNI and/or CTS).

## Authors’ Contributions

M.D.P: drafting/revising the manuscript, study concept and design, analysis and interpretation of data, acquisition of data, and statistical analysis; G.E.G.: drafting/revising the manuscript, acquisition of data; A. C. P.: drafting/revising the manuscript, acquisition of data, interpretation of data. I.V.M.E.: interpretation of data and data analysis. G.P.: drafting/revising the manuscript. B. C. D.: drafting/revising the manuscript, study concept and design. D.R.W.: drafting/revising the manuscript, study design and concept. M.D.D.: drafting/revising the manuscript, study concept and design, interpretation of data, acquisition of data, and study supervision; W.P.T.: drafting/revising the manuscript, study concept and design, interpretation of data, acquisition of data, and study supervision; R.D.D.: drafting/revising the manuscript, study concept and design, interpretation of data, acquisition of data, funding support, and study supervision.

## Conflict of Interest

No authors in this manuscript have any conflict of interest to disclose.

## Authors

**Table:**

Name	Location	Role	Contribution
Michael D. Pridmore	Vanderbilt University Institute of Imaging Science, Vanderbilt University Medical Center, Nashville, Tennessee, USA	Author	Drafting/revising the manuscript, study concept and design, analysis and interpretation of data, acquisition of data, and statistical analysis.
Gabriella E. Glassman	Department of Plastic Surgery, Vanderbilt University Medical Center, Nashville, Tennessee, USA	Author	Drafting/revising the manuscript, acquisition of data.
Alonda C. Pollins	Department of Plastic Surgery, Vanderbilt University Medical Center, Nashville, Tennessee, USA	Author	Drafting/revising the manuscript, acquisition of data, interpretation of data.
Isaac V. Manzanera Esteve	Department of Plastic Surgery, Vanderbilt University Medical Center, Nashville, Tennessee, USA	Author	Interpretation of data and data analysis.
Galen Perdikis	Department of Plastic Surgery, Vanderbilt University Medical Center, Nashville, Tennessee, USA	Author	Drafting/revising the manuscript.
Brian C. Drolet	Department of Plastic Surgery, Vanderbilt University Medical Center, Nashville, Tennessee, USA	Author	Drafting/revising the manuscript, study concept and design.
Douglas R. Weikert	Department of Orthopaedics, Vanderbilt University Medical Center, Nashville, Tennessee, USA	Author	Drafting/revising the manuscript, study concept and design.
Mark D. Does	Department of Biomedical Engineering, Vanderbilt University, Nashville, Tennessee, USA	Author	Drafting/revising the manuscript, study concept and design, interpretation of data, acquisition of data, and study supervision.
Wesley P. Thayer	Department of Plastic Surgery, Vanderbilt University Medical Center, Nashville, Tennessee, USA	Author	Drafting/revising the manuscript, study concept and design, interpretation of data, acquisition of data, funding support, and study supervision.
Richard D. Dortch	Department of Neuroimaging Research, Barrow Neurological Institute, Phoenix, Arizona, USA	Author	Drafting/revising the manuscript, study concept and design, interpretation of data, acquisition of data, funding support, and study supervision.
